# Exploring the Immune-Boosting Functions of Vitamins and Minerals as Nutritional Food Bioactive Compounds: A Comprehensive Review

**DOI:** 10.3390/molecules27020555

**Published:** 2022-01-16

**Authors:** Saikat Mitra, Shyamjit Paul, Sumon Roy, Hriday Sutradhar, Talha Bin Emran, Firzan Nainu, Mayeen Uddin Khandaker, Mohammed Almalki, Polrat Wilairatana, Mohammad S. Mubarak

**Affiliations:** 1 Department of Pharmacy, Faculty of Pharmacy, University of Dhaka, Dhaka 1000, Bangladesh; saikatmitradu@gmail.com (S.M.); shyamjitpaul1998@gmail.com (S.P.); sumonroy10du@gmail.com (S.R.); hridaysd5124@gmail.com (H.S.); 2 Department of Pharmacy, BGC Trust University Bangladesh, Chittagong 4381, Bangladesh; talhabmb@bgctub.ac.bd; 3 Department of Pharmacy, Faculty of Pharmacy, Hasanuddin University, Makassar 90245, Sulawesi Selatan, Indonesia; firzannainu@unhas.ac.id; 4 Centre for Applied Physics and Radiation Technologies, School of Engineering and Technology, Sunway University, Bandar Sunway 47500, Selangor, Malaysia; mayeenk@sunway.edu.my; 5 Department of Nursing, Faculty of Applied Medical Sciences, Taif University, Taif 21944, Saudi Arabia; msmalki@tu.edu.sa; 6 Department of Clinical Tropical Medicine, Faculty of Tropical Medicine, Mahidol University, Bangkok 10400, Thailand; 7 Department of Chemistry, The University of Jordan, Amman 11942, Jordan

**Keywords:** vitamins, minerals, food bioactive compounds, nutrition, immune system

## Abstract

Food components have long been recognized to play a fundamental role in the growth and development of the human body, conferring protective functionalities against foreign matter that can be severe public health problems. Micronutrients such as vitamins and minerals are essential to the human body, and individuals must meet their daily requirements through dietary sources. Micronutrients act as immunomodulators and protect the host immune response, thus preventing immune evasion by pathogenic organisms. Several experimental investigations have been undertaken to appraise the immunomodulatory functions of vitamins and minerals. Based on these experimental findings, this review describes the immune-boosting functionalities of micronutrients and the mechanisms of action through which these functions are mediated. Deficiencies of vitamins and minerals in plasma concentrations can lead to a reduction in the performance of the immune system functioning, representing a key contributor to unfavorable immunological states. This review provides a descriptive overview of the characteristics of the immune system and the utilization of micronutrients (vitamins and minerals) in preventative strategies designed to reduce morbidity and mortality among patients suffering from immune invasions or autoimmune disorders.

## 1. Introduction

The human immune system confers protection against pathogenic microbes and other threats, both internally and externally. A series of anatomical physical barriers, such as the skin, ciliated epithelial cells, the mucous blanket, and mucous membranes, serve as an immunological defense system against foreign substances. When foreign substances manage to bypass these barriers, the immune system reacts rapidly to defend the body against “non-self” particles. The immunological system is comprised of two primary responses: the innate immune system and the adaptive immune system. Regardless of how often an infectious agent is encountered, an innate immune response is triggered. Innate immunity is immediate and relies on receptors that identify foreign particles, also known as pathogen-associated molecular patterns (PAMPs). The innate immune system is comprised of leukocyte responses against pathogens, cell-intrinsic responses against viral infections, and soluble mediators, including complementary proteins, serving as a nonspecific, mutable, and immediate defense system. Conversely, T and B cells are specific to the adaptive immune response. Upon recognizing specific antigens presented by the attacking microbe, these cells generate antibodies that attack and destroy pathogens and facilitate the recruitment of other immune cells that attack the pathogenic factor [1,2].

The nutritional condition of a person plays a significant influence on the efficiency of the immune system. Undernutrition, caused by the inadequate consumption of micronutrients, may impair the body’s ability to support innate immune responses; however, nutritional status does not have the same effects for all illnesses. Although the nutritional status of an individual may predict the clinical progression and prognosis of numerous diseases, including bacterial diarrhea, viral diarrhea, measles, pneumonia, tuberculosis, and others, nutritional status has minimal effects on the clinical progression and consequence of various infectious illnesses, such as viral encephalitis or tetanus, and only a moderate influence on the development of ailments such as HIV and influenza [3].

To date, several experimental studies have reported promising outcomes for the use of vitamins and minerals to boost immune functions. The importance of specific trace elements, vitamins, and minerals for maintaining immunocompetence has been demonstrated in animal models and, in exceptional cases, among humans suffering from nutritional deficits [4]. Important factors include vitamins A, B, C, D, and E, β-carotene, selenium, riboflavin, zinc, and iron [5]. Adding deficient nutrients such as vitamins through dietary changes or supplementation can enhance resistance to infection and recovery of immunological function [6]. Similarly, vitamin-like compounds including choline, carnitine, inositol, and coenzyme Q play crucial roles in immunomodulation [7,8,9,10]. In addition, the antioxidant balance of immune cells must be maintained to protect immune cells against oxidation, and excess quantities of micronutrients may provide functional advantages to the antioxidant system [3,11]. Based on this, the purpose of this review is to describe the attributes of the immune system and the use of micronutrients as preventative measures to reduce morbidities in individuals suffering from immunological invasions or autoimmune diseases.

## 2. Immune System and Immunomodulators

### 2.1. Immune System

Nonpathogenic and pathogenic microorganisms, allergens, and other toxic chemicals can disrupt the maintenance of homeostasis in the host body. To counter these invasive factors, the host defense system exerts a protective mechanism referred to as the immune system [12]. The immune response depends on the identification of a toxin or pathogen through structural characteristics that distinguish them as invasive and differentiated from the host cells. Selectivity against foreign pathogens and toxins is crucial for the ability of the host immune system to eradicate the invasive factor without injuring host tissues [13]. Mechanisms used to identify microbial, poisonous, or allergenic structures can be divided into two categories. Initial responses are performed by the innate immune system, and the recognition markers utilized by this system are extensively expressed on a wide variety of cell types. The innate immune system can activate rapidly when invasive pathogens or toxins are identified, representing the immediate host response to the detection of foreign particles [14]. The innate immune response represents a nonspecific immune response that can be found in all multicellular organisms and constitutes the initial host defense against various pathogens. This response is crucial for ensuring cellular homeostasis, removing a wide range of pathogens, and supporting the activation of the adaptive immune response [15]. The innate immune system is comprised of both cell-mediated and humoral components and physical and chemical barriers. The main effectors of innate immunity include mast cells, macrophages or monocytes, the complement system, natural killer (NK) cells, basophils, and neutrophils [16].

The adaptive immune response is molded by the detection of antigens and comprises several cells with specificity for a particular microorganism, allergen, or toxin. An adequate adaptive immune response requires sufficient immune cell proliferation to mount an effective response to the detected toxin or microbe. Adaptive responses are typically activated shortly after the activation of the innate response [17]. In this respect, the T and B lymphocytes are the major components of the adaptive immune system. In contrast with the specific pathogen receptors used to detect pathogens by the innate immune system, the adaptive immune system boasts an enormously diverse and randomly produced receptor repertoire, which allows the adaptive immune system to recognize a wide variety of antigens. However, this diversity comes at a price, including the risk of autoimmune disease, in which receptors expressed by B and T cells through the random gene rearrangement process recognize self-proteins, such as myelin and insulin. Intricate tolerance mechanisms must be implemented to eliminate or regulate self-reactive immune cells. Another limitation is the lag time required to generate an adaptive immune response following the initial exposure to a pathogen. The clonal proliferation of unique antigen-specific T and B cells can require up to 5 days before the adaptive immune response is sufficiently robust enough to eradicate infectious disease [18].

### 2.2. Immune Activation in Response to Non-Self-Antigens

Antigens are physical and chemical entities that are capable of eliciting particular immune responses in the body. When an unfamiliar pathogenic microbe is detected within the host, the innate immune system may be activated, resulting in the development of adaptive immunity against the pathogenic agent. In vertebrates, Nod-like receptors (NLRs), Toll-like receptors (TLRs), C-type lectin receptors (CLRs), and RIG-I-like receptors (RLRs) are among the pattern recognition receptors (PRRs) used to detect microbial invasions [19]. PRRs are expressed in the cytoplasm and on the cell surface. TLRs are membrane receptors that identify a wide range of microbial components, including microbe-specific nucleic acids, glycolipids, lipoproteins, and proteins, performing pivotal roles in both the adaptive and innate immune responses. By contrast, NLRs recognize bacterial elements found in the cytoplasm, RLRs detect viral replication-induced double-stranded RNA, and CLRs recognize fungal and bacterial sugar moieties [20]. Non-immune cells, such as endothelial cells, epithelial cells, and fibroblasts, can also detect pathogens and trigger innate immunological responses in infected tissues, resulting in the production of a wide range of chemokines and cytokines, in addition to the accumulation and activation of immune cells and leukocytes [21]. In addition to dendritic cells (DCs), which migrate from contaminated tissues to a regional lymph node, where they activate cognate T cells, other immune cells express microbe-derived peptides and the major histocompatibility antigen on their cell surfaces, resulting in the establishment of adaptive immunity and the development of antigen specificity [19].

### 2.3. Regulation of Immune Responses

Immune regulation refers to the process of balancing immune activation and repression to ensure an effective immune response without damaging the host and reflects a comprehensive and complex process in the human body. T regulatory cells (Tregs), chemokines, cytokines, antibodies, and their receptors all influence immune responses. Foxhead box protein P3 (FOXP3^+^) Tregs are considered to be essential players in the maintenance of appropriate immunological regulation and control of the immune system through both contact-dependent and contact-independent mechanisms. These mechanisms are regulated by FOXP3 and include the inhibition of T effector cell proliferation and the production of inflammatory cytokines [22].

T cells can be classified into two types: T4 cells, also known as CD4, inducer, or T helper (Th) cells; and T8 cells, also known as CD8 cells or cytotoxic T cells. CD4 (Th or inducer cells) lymphocytes are part of the immune system’s regulatory mechanism. Macrophages activate antigen-specific T4 cells through direct cell-to-cell contacts after an antigen has been digested. After activation, the T4 cells produce IL-2, which stimulates other T4 cells, in addition to the cytotoxic and delayed hypersensitivity T8 cells. Stimulated T4 cells can also generate lymphokines, which activate B cells. Suppressor T8 cells regulate the development and function of T4 cells through downregulation or negative feedback [23]. Suppressor T8 cells also limit antibody synthesis by plasma cells, reduce cytotoxic T8 cell activation, and play roles in transplanted tissue tolerance, among other functions.

Cytokines can both stimulate and suppress the immune system and play vital roles in the control of immunological responses. Both immune and non-immune cells generate extracellular vesicles, which perform pivotal roles in immune regulation [24] by stimulating or inhibiting the immune system and driving the pathophysiology of inflammatory, autoimmune, and viral diseases. Extracellular vesicles might thus be utilized to regulate the immune system as a therapeutic agent.

### 2.4. Immunomodulators

Immunomodulators are naturally derived compounds and metabolites produced by healthy immune systems that maintain the equilibrium of the body. Immunomodulators are biological or synthetic drugs that can stimulate, inhibit, or regulate the components of the immune system or interfere with the immune system’s functions [25]. In clinical practice, immunomodulators are classified into three main categories: immunostimulating agents, immunosuppressive agents, and immunoadjuvants. Immunoadjuvants are substances that boost the effectiveness of vaccines, which provide an immune-stimulating effect [26].

#### 2.4.1. Immunostimulators

Immunostimulators, also known as immunostimulants, are substances that stimulate the immune system by activating or increasing the activity of one or more components. Immunostimulators boost the body’s immunity against allergens, infections, cancer, and autoimmune responses. Within this context, levamisole is a synthetic anthelmintic and immunomodulatory agent that can be administered orally. Levamisole combined with fluorouracil reduces recurrence and mortality risk by one-third among individuals with surgically resected stage C colorectal cancer [25]. The mechanism through which levamisole attacks the immune system in humans is complex and incompletely understood; however, the bulk of existing in vitro and in vivo evidence suggests that levamisole has immune-restorative properties that are able to strengthen a weakened host immune system without over stimulating a healthy immune system. Granulocytopenia is the most significant side effect associated with levamisole.

Multiple myeloma and erythema nodosum leprosum patients are often treated with thalidomide, which is an orphan medication used to treat Kaposi sarcoma, mycobacterial infections, brain malignancies, HIV-related wasting, lupus, myelofibrosis, Crohn’s disease, aphthous ulcers, leprosy, and graft-versus-host disease [16]. Pregnant women or those who may get pregnant should avoid thalidomide use due to severe side effects to the fetus.

Lenalidomide is thalidomide analog with anti-angiogenic and immunomodulatory effects. Lenalidomide has been authorized by the US Food and Drug Administration (FDA) for the treatment of individuals with transfusion-dependent anemia caused by low- or intermediate-risk myelodysplastic syndromes, regardless of the presence of other chromosomal abnormalities [16].

The most significant immunostimulant is the Bacille Calmette-Guérin (BCG) vaccine, which can also be used in a topical form to treat recurrent superficial bladder cancer. The possible mechanisms of action include a human leukocyte antigen (HLA)-restricted T cell response due to the BCG-stimulated expression of HLA class II antigens by tumor cells. The level of IL-2 in the urine is directly linked to treatment response [25]. Topical BCG is being tested for the treatment of melanomas and head and neck cancers. Interferons (IFNs, α, β, γ, and IL-2) and other immunostimulants have key immunomodulatory properties.

#### 2.4.2. Immunosuppressants

Immunosuppressants are drugs that suppress the immune system, which can be used to treat autoimmune disorders, hypersensitive immunological reactions, and immune pathology linked with infections, in addition to regulating pathological immune responses following organ transplantation. Immunosuppressive medications that are commonly used in transplantation include calcineurin inhibitors, biologicals (antibodies), antimetabolic/anti-proliferative agents, and glucocorticoids [16].

Glucocorticoids cause lymphocyte redistribution, resulting in a rapid but temporary reduction in lymphocyte numbers in the peripheral circulation. Cyclosporines are calcineurin inhibitors with specificity. Cyclosporines suppress the immune response by decreasing the synthesis of IL-2, which prevents the activation and proliferation of CD4^+^ and CD8^+^ T cells [27]. Similar to cyclosporines, tacrolimus suppresses T cell activation by blocking calcineurin. Mycophenolate mofetil is a prodrug that is rapidly hydrolyzed into mycophenolic acid (MPA), a selective, non-competitive, reversible inhibitor of inosine monophosphate dehydrogenase (IMPDH), an essential enzyme involved in the de novo synthesis of the guanine nucleotide, which B and T lymphocytes rely heavily on for cell proliferation, in contrast with other cell types that employ salvage mechanisms. Therefore, MPA preferentially suppresses lymphocytic proliferation and other activities, including cellular adhesion, migration, and antibody production [13]. FTY720 (immunomodulator drug), unlike other immunosuppressive drugs, works through lymphocyte homing, sequestering host lymphocytes into lymph nodes and Peyer’s patches in a selective and reversible manner, preventing their circulation.

### 2.5. The Importance of Immunomodulators in Current Clinical Practice

Immunomodulators are currently the best weapons for treating many immune-related disorders and can be used to enhance immune function when serious ailments trigger a poor immune response. Immunosuppressive agents are often used to treat various autoimmune disorders in clinical practice, such as the use of immunosuppressive agents to prevent graft rejection. Immunosuppressive cyclosporines are commonly used to treat rheumatoid arthritis and psoriasis and to support the success of heart transplantation [16]. Monoclonal antibodies and biological response modifiers play significant roles in treating illnesses such as cancer, injuries, autoimmune disorders, cardiovascular diseases, and severe infections [25]. These drugs are typically more effective when used in combination with other medications (e.g., antibiotics, cytotoxics, and other cytokines). Immunomodulators are also widely used in immunotherapy to treat individuals with immune-related disorders.

## 3. Immune-Boosting Foods

The best method for obtaining the micronutrients required to improve immune system functions is through a proper diet, including the consumption of vegetables and fresh fruits [28]. For example, elderberries contain antioxidants and may help to reduce inflammation, whereas selenium and B vitamins are abundant in button mushrooms. Other foods with high selenium contents include sardines, garlic, broccoli, Brazil nuts, tuna, and barley. Zinc can be obtained from lean meats, wheat germ, oysters, crabs, poultry, chickpeas, yogurt, and baked beans, among other foods [29]. Heme is available from lean poultry and shellfish and represents the most readily absorbed source of iron. Other healthy sources of iron include broccoli, kale, and beans. Many vitamins that aid immunity can be found in foods or are added to foods, such as folic acid, which is added to several cereals, breads, pastas, and other whole grains. The natural form of folate is found in beans, peas, and green leafy vegetables [29,30]. Baked potatoes, lean chicken breast, chickpeas, cold-water fish, such as tuna, and bananas are all high in vitamin B_6_. Green leafy vegetables, including spinach and kale, in addition to citrus fruits, contain vitamin C, which is also abundant in brussels sprouts, bell peppers, strawberries, and papaya [31]. Most people can obtain sufficient amounts of vitamin C without requiring supplements, as vitamin C–rich foods are easily accessible. Vitamin E is abundant in hazelnuts, peanuts, almonds, and sunflower seeds, in addition to spinach and broccoli. Choosing a range of colorful foods ensures the acquisition of sufficient dietary vitamin A amounts [3].

## 4. Insights into the Key Roles Played by Vitamins and Minerals as Immunomodulators

The nutritional benefits of vitamins and minerals depend on not only the amount of nutrients consumed, but also on how readily they are absorbed into the body. For this, food processing is one of the most important factors affecting bioavailability since it may either increase or decrease the bioaccessibility of nutrients and bioactive compounds [32]. In fact, processing methods are getting more sophisticated and diversified to meet the growing demands for high-quality foods, with nonthermal processing emerging as a good method for extending shelf-life and product quality while also preserving functional and nutritional properties. Therefore, efforts should be made during processing to mitigate the influence of applied technology on vitamins and minerals, and to be more favorable if nutritional quality is measured in terms of both stability and bioaccessibility [33].

For optimum physiological functioning to occur, adequate nutrition must be provided in a balanced manner in order to avoid potentially harmful interactions, particularly when medications are supplied at pharmacological concentrations. In this respect, several nutrients work together to enhance digestive function and absorption by complementing one another. In contrast, some may obstruct these processes and compete for absorption, while others may be needed to work in tandem to facilitate metabolism, potentially affecting a variety of biochemical processes [34]. The human body has a lot of synergistic and antagonistic functionalities that need to be taken into account, especially in the health and research fields, and where nutrient-related confounding factors also need to be taken into consideration [35]. Below are the key roles played by vitamins and minerals as immunomodulators.

### 4.1. Vitamin A

The recommended daily allowance of vitamin A is 900 micrograms (µg) for women and 700 µg for men. The richest food sources of vitamin A include dairy products, fish, liver, and fortified cereals; the top sources of provitamin A include broccoli, carrots, squash, and cantaloupe [36]. Vitamin A and β-carotene are structurally interrelated, and the liver converts β-carotene to vitamin A. One molecule of β-carotene yields two molecules of vitamin A. When we look at the two molecules side by side, we can see that vitamin A is remarkably similar to half of the β-carotene molecule. β-Carotene is a plant pigment that is responsible for the vibrant colors of red, orange, and yellow vegetables. Β-Carotene is classified as a provitamin A carotenoid, which means that it can be converted by the body into vitamin A [37].

Vitamin A deficiency causes illnesses and deaths worldwide, particularly among women, children, and infants in low-income countries. Approximately 253 million children are at high risk of experiencing immunodeficiency due to vitamin A deficiencies [38]. On the other hand, vitamin A supplementation in individuals with hypovitaminosis A decreases morbidity and mortality rates due to diarrheal and measles diseases [39,40]. Similarly, regular supplementation with high-dose vitamin A could decrease both morbidity and mortality among infants delivered by HIV-positive mothers [41], in addition to reducing diarrhea-associated morbidity among HIV-positive children following hospital release for acute lower respiratory tract infections [42].

Vitamin A exists in three main forms: retinoic acid (RA), retinol, and retinal, and RA has demonstrated remarkable biological activities [43]. Experimental work by Rampal et al. [44] explored the role of RA in human dendritic cells (hDCs) and CD4^+^ T cell responses. DCs primed with RA (RA monocyte-derived DCs) increased the expression of C-C motif chemokine receptor 9 (CCR9)^+^ T cells, which express large amounts of IFN-γ under Th1/Th17 circumstances and also promote the production of IL-17^+^ T cells. In addition, RA monocyte-derived DCs suppress IL-9 production and increase IFN expression in T cells when stimulated with transforming growth factor (TGF)-1 or IL-4. RA treatment reduces the T cell production of IFN and IL-17 in experiments with naive CD4^+^ T cells stimulated under Th1/Th17 conditions in the absence of DCs. These findings indicated that RA, instead of acting as an anti-inflammatory agent, may sustain or worsen intestinal inflammation under inflammatory conditions [45]. RA significantly affects macrophage activity by regulating cytokine release, including IL-1, IL-6, IL-12, tumor necrosis factor (TNF)-α, and nitric oxide (NO). Moreover, T cell–macrophage co-cultures treated with RA show reduced IFN-γ production and increased IL-4 secretion by T cells, demonstrating that RA can influence Th1/Th2 differentiation pathways. RA also inhibits the expression of IL-12, which is thought to stimulate IFN production by T cells [46].

RA inhibits intestinal DC inflammation by targeting nuclear factor kappa B (NF-κB), which can induce both anti- and pro-inflammatory actions. RA combined with IL-4 promotes the release of suppressor of cytokine signaling 3 (SOCS3), which suppresses the production of pro-inflammatory cytokines, such as TNF-α and IL-12p70 [47]. When RA is combined with IL-15, the c-Jun N-terminal kinase (JNK) pathway becomes activated, increasing the production of pro-inflammatory cytokines by DCs, including IL-12p70 and IL-23 [48].

### 4.2. Vitamin B_1_

Vitamin B_1_, also known as thiamin or thiamine, is a water-soluble vitamin that may be obtained from foods, dietary supplements, and medications. The recommended daily amount of vitamin B_1_ is 1.1 mg for women and 1.2 mg for adult men. The release of intracellular adhesion molecules (ICAMs) by thiamine–immune cell interactions is mediated by hemin-dependent oxygenases. ICAMS bind to cells expressing different integrins, regulating immune cell localization. Thiamine exerts antioxidant actions to protect cell surface sulfhydryl groups found on neutrophils from oxidative damage. In this regard, thiamine inhibits the oxidative-stress-mediated stimulation of NF-κB and the formation of pro-inflammatory cytokines in macrophages [49].

Thiamine deficiency has been associated with neuroinflammation, T cell infiltration, and the overexpression of pro-inflammatory cytokines (IL-1, IL-6, and TNF-α). Additionally, thiamine deficiencies upregulate the expression of CD40 and CD40L by microglial cells and astrocytes, inducing neuronal death [50]. Within this context, Bozic and coworkers reported that benfotiamine (synthetic thiamine) significantly reduces the inflammatory response in lipopolysaccharide (LPS)-induced BV-2 microglia. LPS stimulation activates pro-inflammatory functions in microglial cells in vitro. Moreover, benfotiamine decreases the expression of inducible NO synthases (iNOS) and NO while increasing the expression of cyclooxygenase-2 (COX-2), heat shock protein 70, IL-6, TNF-α, and NF-κB [51]. Thiamine also suppresses the pro-oxidative activity of microglial cells, suggesting that thiamine may be used to treat neurodegenerative diseases. Similarly, LPS increases the production of thromboxane 2, leukotrienes, prostacyclin, prostaglandin E2, NF-κB, COX-2, and iNOS and induces macrophage cell death in the RAW264.7 murine macrophage cell line, and these effects could all be suppressed by the presence of benfotiamine [52].

### 4.3. Vitamin B_2_

Vitamin B_2_, also known as riboflavin, serves as a vital cofactor for several enzymes involved in energy metabolism [53]. For men aged 19 and over, the recommended daily amount (RDA) of vitamin B2 is 1.3 mg per day, while for women, it is 1.1 mg per day. Women should consume 1.4 mg per day throughout pregnancy and 1.6 mg per day during breastfeeding. Bacterial metabolites of vitamin B_2_, when combined with the non-classical major histocompatibility complex (MHC) class I-related protein (MR1), activate innate mucosal-associated invariant T (MAIT) cells [54], which produce IFN-γ and IL-17 and play pivotal roles in mucosal defense and inflammation in the gut [55].

Riboflavin is a potent anti-inflammatory modulator with antioxidant and anti-tumor properties that increases the phagocytic activity of macrophages [56]. Riboflavin affects numerous metabolic processes in a roundabout way and is essential for the appropriate functioning of a number of systems, including the immune system. The effects of riboflavin at doses from 3 to 531 nM have been examined against the activity and proliferation of RAW 264.7 murine macrophages, which are a type of immune-competent cell. Riboflavin deficiency impairs macrophage activity and survival, reducing their capacity to mount an immunological response. Within 4 days of growth in media with low riboflavin contents, RAW 264.7 cells showed signs of riboflavin deficiency, including slowed cell growth and the induction of apoptosis, which released lactate dehydrogenase. Riboflavin starvation prevented respiratory bursts and significantly hindered phagocytosis, suggesting that riboflavin deficiency may impact the immune system, decreasing the body’s ability to mount an adequate host immunological response [57].

### 4.4. Vitamin B_3_ (Niacin, Nicotinic Acid, and Nicotinamide)

The recommended daily amount of vitamin B_3_ is 14 mg for women and 16 mg for adult men. Vitamin B_3_ can activate the innate immune system by up to 1000-fold the normal level, making it an effective initial line of defense against pathogens. At large dosages, nicotinamide can protect the body against *Staphylococcus aureus* infections. Nicotinic acid supplementation has been found to reduce inflammation via monocytes in models of atherosclerosis [58,59].

To explore how niacin affects blood vessel inflammation in vivo and in vitro and identify the niacin-associated lipid regulatory mechanism, niacin was administered to guinea pigs fed a high-fat diet, resulting in reduced levels of inflammatory factors (IL-6 and TNF-α) in plasma, decreased CD68 and NF-κB p65 protein expression in the arterial wall, and reduced oxidative stress. In oxidized low-density lipoprotein (oxLDL)-stimulated human umbilical vein epithelial cells (HUVECs) and THP-1 macrophages, niacin reduced IL-6 and TNF-α secretion, suppressed NF-κB p65 and notch 1 protein production, and reduced HUVEC apoptosis. Furthermore, niacin reduced lipid deposition in the artery wall, raised high-density lipoprotein cholesterol (HDL-C) and apolipoprotein A (ApoA) levels in plasma while decreasing triglycerides (TG) and non-HDL-C levels, and elevated the mRNA expression level of cholesterol 7-hydroxylase A1 in the guinea pig liver. The findings indicate that niacin reduces vascular inflammation in vivo and in vitro via NF-κB signaling pathway inhibition [60]. Another experimental study reported that niacin decreased the levels of IL-1α, IL-6, and TNF-α in alveolar macrophages exposed to LPS. NF-κB activation was also reduced by niacin through the inhibition of NF-κB p65 phosphorylation and nuclear factor-1 B (NFIB) phosphorylation. In addition, the inhibition of hydroxycarboxylic acid receptor 2 (HCA2) prevented the niacin-induced production of pro-inflammatory cytokines. These results indicated that niacin inhibited the production of pro-inflammatory cytokines by LPS-mediated alveolar macrophages, which may have been mediated by HCA2 [61].

### 4.5. Vitamin B_5_ (Pantothenic Acid)

The recommended amount of pantothenic acid for adults is 5 mg per day. Vitamin B_5_, also known as pantothenic acid, is found in both animals and plants, and is available in a variety of foods such as vegetables, meat, cereal grains, eggs, legumes, and milk. Examination of the antibacterial and pro-inflammatory effects of pantothenic acid in macrophages infected with the *Mycobacterium tuberculosis* strain H37Rv revealed the in vivo therapeutic value of pantothenic acid for patients with tuberculosis. Vitamin B_5_ (VB_5_) was used to treat H37Rv-infected mice to determine whether VB_5_ promotes H37Rv clearance from the lungs and whether VB_5_ regulates inflammatory cells. VB_5_ increased phagocytosis and upregulated the inflammatory response in macrophages infected with H37Rv. Research findings indicated that oral administration of VB_5_ to mice 1, 2, and 4 weeks after infection caused reduction in the H37Rv colony-forming units detected in the lungs. The proportion of macrophages was also regulated, and CD4^+^ T cells were stimulated to produce IFN-γ and IL-17; however, the percentages of polymorphic nuclear neutrophils and CD4^+^ and CD8^+^ T cells were unaffected by VB_5_ administration. VB_5_ substantially suppressed the development of *M*. *tuberculosis* b modulating both innate and adaptive immunity [62].

Dexpanthenol (a vitamin B_5_ derivative) substantially alleviates pulmonary edema in mice, preventing neutrophil accumulation in the lungs and enhancing superoxide dismutase (SOD) levels [63]. Dexpanthenol also decreased TNF-α levels, reduced the total oxidant status, and reduced the oxidative stress index in endometriosis patients [64]. After inducing necrotizing enterocolitis, dexpanthenol decreased intestinal damage, increased antioxidant enzyme (SOD) and glutathione (GSH) activity, and induced the production of pro-inflammatory cytokines (IL-1 and TNF-α) [65].

### 4.6. Vitamin B_6_ (Pyridoxine)

The recommended daily allowance of vitamin B_6_ for adults 50 and younger is 1.3 mg. Deficiency in vitamin B_6_, also known as pyridoxine, results in decreased antibody production and increased IL-4 levels. Mice fed a pyridoxine-deficient diet exhibit altered T cell responses, including the suppression of T cell proliferation, decreased IL-2 levels, increased IL-4 levels, and the altered expression of transcription factors, including T-bet and SOCS-1 [66]. Vitamin B_6_ deficiencies in young grass carp (*Ctenopharyngodon idella*) results in decreased anti-inflammatory cytokine production (TGF-β, IL-4, IL-10, IL-11, and IL-13) and increased pro-inflammatory cytokine production (IL-1, IL-6, IL-8, IL-12, IL-15, and IL-17) [67]. Weaned Rex rabbits fed with a vitamin B_6_ supplemented diet revealed significantly increased levels of IL-6 and IFN mRNA expression in the spleen (*p* < 0.05). Furthermore, vitamin B_6_ significantly enhanced the number of M cells in the appendix (*p* < 0.05).

**Figure 1:**
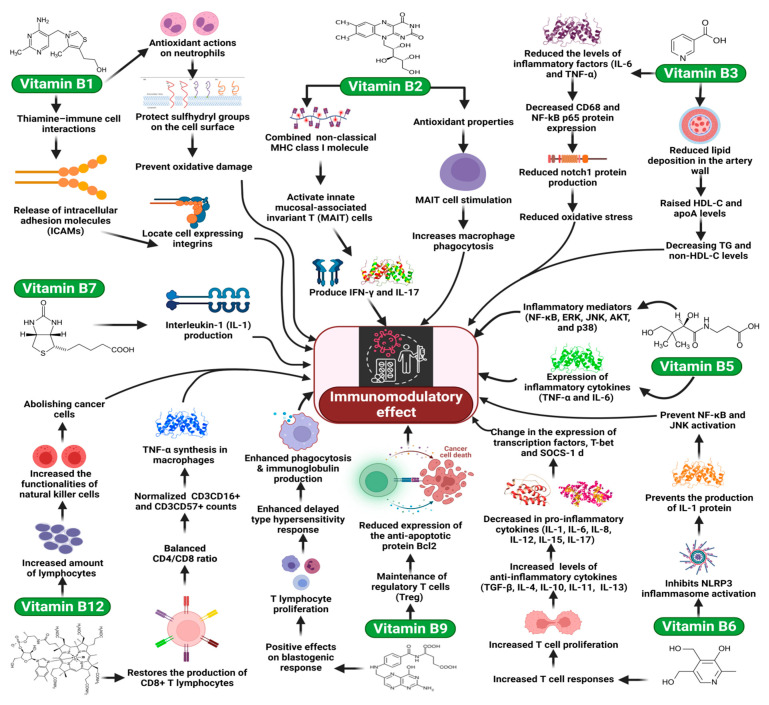
The key role of vitamin B as immunomodulators.

Vitamin B_6_ was found to affect the immunological performance of rabbits, and vitamin B_6_ supplementation was recommended in weaning three-month-old developing Rex rabbits at a dose of 10–20 mg/kg [68]. Another study by Zhang et al. found that vitamin B_6_ inhibits NOD-, LRR- and pyrin domain-containing protein 3 (NLRP3) inflammasome activation, which in turn prevents the production of IL-1. Both pyridoxal (PL) and pyridoxal 5′-phosphate (PLP) suppressed the expression of cytokine genes and inhibited the processing of caspase-1 and the subsequent production of mature IL-1 and IL-18 in LPS-primed macrophages, indicating that they were inhibiting NLRP3-dependent processing. When administered to peritoneal macrophages, PLP but not PL significantly decreased the generation of mitochondrial reactive oxygen species (ROS) (Figure 1). Importantly, PL and PLP decreased IL-1 production in mice when LPS and ATP were combined or when LPS was used alone. In addition, both PL and PLP administration protected mice against fatal endotoxins. Taken together, these results indicate the unexplored anti-inflammatory properties of vitamin B_6_, which might be useful for the prevention of inflammation-related illnesses caused by the NLRP3 inflammasome [69].

### 4.7. Vitamin B_7_ (Biotin)

Vitamin B_7_, also known as biotin, is a water-soluble B complex vitamin that plays a pivotal role in the development of chronic inflammatory conditions [70]. The recommended amount of biotin for adults is 30 mg per day. Kuroishi et al. explored the effects of biotin status on nickel (Ni) allergies in mice by assessing the modulation of IL-1 production. The authors found considerably increased IL-1 production by splenocytes from biotin-deficient mice compared with splenocytes from biotin-sufficient mice. Biotin-deficient mouse macrophage J774.1 cells produce substantially higher levels of IL-1 mRNA and protein than biotin-sufficient cells. In vitro, biotin supplementation prevents the induction of IL-1 production, and the in vivo addition of biotin to drinking water resulted in a dose-dependent reduction in ear edema in both biotin-sufficient and biotin-deficient mice. These findings indicated that biotin deficiency impacts Ni allergy in mice during the elicitation phase via the elevation of IL-1 production, suggesting that biotin supplementation may have therapeutic benefits for the treatment of human metal allergies [71].

### 4.8. Vitamin B_9_ (Folate)

Vitamin B_9_, also known as folate, is a water-soluble vitamin that can be obtained in two forms: dietary folate, which is naturally available in juices and citrus fruits, among other foods; and folic acid, which is used as a dietary fortification and delivered via supplements. The recommended daily amount of folate is 400 µg for adults, whereas women who are considering pregnancy or may be pregnant should take 400 to 1000 µg of folic acid per day. In vitro, in vivo, and human studies suggest that folate supplementation is correlated with lower infection rates, positive effects on blastogenic responses and T lymphocyte proliferation, delayed hypersensitivity responses, increased phagocytosis, and upregulated immunoglobulin production. However, folate does not appear to affect the function of NK cells. The negative consequences of folate deficiencies on immunological function are likely mediated by abnormalities in DNA and RNA synthesis or methyl metabolism, which are both affected substantially by folate availability [72]. Researchers observed that folate plays an integral role in Treg maintenance, both in vitro and in vivo. Treg cells that express high levels of folate receptor 4 show improved survival in response to folate administration. In an in vitro study, Treg cells could be distinguished from naive T cells under conditions of low folate levels, but they were unable to survive in this environment. The reduced expression of the anti-apoptotic protein Bcl2 was associated with poor Treg cell survival under folate-deficient conditions, independent of IL-2 (Figure 1). Dietary folate deficiency is associated with decreased Treg cells in the small intestine, which is an important location for dietary folate absorption. These results establish a novel connection between food and the immune system, which may have implications for the maintenance of immunological homeostasis in the gastrointestinal tract [71].

### 4.9. Vitamin B_12_ (Cobalamin)

The recommended amount of cobalamin for adults is 2.4 µg per day. Vitamin B_12_ deficiency results in the downregulation of lymphocytes and affects the functionalities of NK cells, which are instrumental in abolishing cancer cells [73]. More precisely, patients with vitamin B_12_-deficient anemia have a smaller proportion of CD8^+^ T cells than the general population. Compared with healthy individuals, the proportions of CD4^+^ lymphocytes were significantly higher in patients with vitamin B_12_ deficiency, resulting in an abnormally high CD4^+^/CD8^+^ ratio. In people with vitamin B_12_ deficiency, NK cell activity is significantly reduced, and splenic NK activity is also reduced in rats fed a vitamin B_12_-deficient diet; however, no significant impacts on NK activity were observed in the axillary nodes or thymus [73,74].

Intramuscular administration of vitamin B_12_ (in the form of methylcobalamin) to patients who have recently been diagnosed with vitamin B_12_ deficiency completely restores production of CD8^+^ T lymphocytes, returns the CD4^+^/CD8^+^ ratio back to normal levels, and restores CD3CD16^+^ and CD3CD57^+^ counts (both of which have high NK cell activity) (Figure 1), which results in restored NK cell activity [73]. Conversely, vitamin B_12_ deficiency or supplementation had no effects on immunoglobulin levels in the blood [73]. These findings suggest that CD8^+^ T cells can be upregulated, and the CD4^+^/CD8^+^ ratio can be normalized by intramuscular cyanocobalamin injections in patients with pernicious anemia and low vitamin B_12_ levels (three to ten times lower than the reference level) [75].

Similarly, findings showed that TNF-α production has increased in the spinal cords vitamin B_12_-deficient rats, whereas TNF-α synthesis was elevated in the macrophages of vitamin B_12_-deficient mice [76]. Furthermore, due to glycoprotein 130 (gp130) dysregulation, vitamin B_12_-deficient animals demonstrated reduced IL-6 production [76].

### 4.10. Vitamin C

The daily dose of vitamin C recommended for adults is 65 to 90 mg. Vitamin C is a water-soluble antioxidant found in plasma and cells. In addition to roles in metabolic functions, vitamin C appears to contribute to the maintenance of immune homeostasis. Recent in vitro experiments examined the inhibitory effects of vitamin C on the expression of pro-inflammatory mediators, including IL-6 and TNF-α, in adult blood cells. Vitamin C has been proposed to act as a potential modulator that prevents an overexuberant immune response, such as in patient groups at risk of developing systemic inflammatory response syndrome, including newborns [72]. Vitamin C may also influence LPS-induced gene expression in human macrophages via NF-κB [77]. In an in vitro study, vitamin-C-pretreated murine IgM/CD40-activated B cells showed a low level of apoptosis induction in a dose-dependent manner, whereas lower vitamin C concentrations supported the antioxidant properties of activated B cells, without affecting cell proliferation or the expression of distinct surface molecules, such as CD80 and CD86 [78]. In a clinical study involving healthy male university students, vitamin C resulted in substantial increases in IgA and IgM levels in blood [79].

Research findings showed that platelet aggregation inhibits tumor cell migration, and the in vitro administration of vitamin C enhanced the capacity of NK cells to kill tumor cells [80]. In addition, leukocytes, including lymphocytes, actively accumulate vitamin C in the presence of a concentration gradient, highlighting the vitamin-C-dependent functional and developmental characteristics of immune cells. Vitamin C is an antioxidant with notable effects on both the innate and adaptive immune responses and is required for the metabolism of microorganisms. Furthermore, vitamin C has been shown to limit the development of several bacterial species. However, the presence of this vitamin can induce oxidative stress in other bacterial species, which may result in the inhibition of bacterial growth [79].

### 4.11. Vitamin D

Vitamin D intake should be 400 international units (IU) for children under the age of 1 year, 600 IU for persons between the ages of 1 to 70, and 800 IU for those beyond 70. Vitamin D was postulated to play multifunctional roles in the immune system following the identification of the vitamin D receptor (VDR) in macrophages, DCs, and activated T and B lymphocytes, in addition to the report that these cells produce 25-hydroxyvitamin D 1-alpha-hydroxylase (encoded by *CYP27B1*) [81].

### 4.12. The Role of Vitamin D in Innate Immune Response

The phagocytic activity of macrophages and NK cell functionalities are stimulated by treatment with VDR ligands whereas the stimulatory abilities of monocytes and macrophages are decreased, as indicated by the reduced surface expression of MHC-II and co-stimulatory molecules [82]. Vitamin D modulates immune functions via the regulation of nuclear transcription factors, including nuclear factor of activated T cells (NFAT) and NF-κB, or through the direct binding to vitamin-D-responsive sites on cytokine gene promoters [83]. Intracellular IL-1, IL-6, TNF-α, IL-8, and IL-12 expression in monocytes is inhibited by vitamin D [84]. The TNF-α gene promotor contains VDR-responsive regions, whereas the IFN-γ gene promoter contains a negative transcriptional regulatory element that binds vitamin D. VDR monomers bind to the repressive complex in the IFN-γ promoter, competing with the nuclear factor angiotensin type 1 (AT1) to control granulocyte-macrophage colony-stimulating factor (GM-CSF). In addition, vitamin D inhibits NF-κB activation by boosting inhibitor of NF-κB (IκBα) expression and interfering with the binding of NF-κB-regulated genes (IL-8, IL-12, etc.) [85].

Studies have focused on the impacts of vitamin D on the maturation, differentiation, and migration of antigen-presenting DCs, revealing that the increased expression of MHC class II, CD40, CD80, and CD86 in DCs inhibits differentiation, maturation, and immunostimulatory abilities [86]. The active form of vitamin D, 1,25(OH)2 D3, suppresses DC maturation by downregulating IL-12, upregulating IL-10, and reducing the antigen-presenting capabilities of DCs, decreasing the stimulation of T cell proliferation and generation, and promoting a Th2 response [4]. Furthermore, the active form of vitamin D promotes the production of various endogenous antimicrobial peptides with a wide range of activities against viruses, bacteria, and fungi [87]. The activation of TLR ligands in response to innate immunity results in a direct antimicrobial infection response by polymorphic nuclear cells, monocytes, and macrophages [88]. ROS and antimicrobial peptides such as cathelicidins are produced by the immunological response. Functional vitamin-D-responsive regions are found in the gene promoter of human antimicrobial cathelicidin peptides, which are transcribed in response to the vitamin D treatment of human keratinocytes, monocytes, and neutrophils. Moreover, upregulation of VDR and CYP27B1 expression by TLRs leads to the intracrine, vitamin-D-dependent production of cathelicidins, and enhanced macrophage microbicidal activity. Cathelicidins also promote the release of IL-6, IL-10, and IL-18, in addition to the expression of the epidermal growth factor receptor, and enhanced neutrophil, monocyte, macrophage, and T cell chemotaxis, along with the proliferation and migration of keratinocytes [82]. Research findings showed that vitamin D activates autophagy and facilitates the co-localization of mycobacteria and antimicrobial peptides inside autophagosomes, resulting in an increase in bacterial elimination rate. In addition, vitamin D suppresses the expression of TLR2 and TLR4 on monocytes, resulting in a PAMP hyporesponsive state [89].

### 4.13. The Role of Vitamin D in Adaptive Immune Response

Vitamin D exerts immunomodulatory effects on the adaptive immune system through its direct influence on T lymphocytes, particularly Th cells, and B lymphocyte proliferation, differentiation, and apoptosis [84]. Several lines of evidence suggest that vitamin D can inhibit the effects of the adaptive immune system [90], including suppression of Th1 cell formation and reducing IFN-γ, IL-2, and IL-12 production, which are essential for Th cell development [91,92]. Vitamin D also suppresses the growth and activity of Th17 cells, including the production of IL-17 and IL-21, mediated by the reduced secretion of IL-23 and IL-6. In this respect, reduced IFN-γ levels suppress T cell recruitment, whereas reduced IL-2 levels suppress T cell growth. Additionally, inhibition of IL-12 production supports Th2 cell growth, increasing IL-4, IL-5, and IL-10 production and further suppressing Th1 cells, thus shifting the balance toward a Th2 phenotypic profile [93,94]. Furthermore, the Th17 cell line produces IL-17, and has been implicated in the development of several autoimmune diseases; however, several experimental studies suggest that vitamin D_3_ suppresses Th17 formation and activity by blocking NFAT and runt-related transcription factor 1 (RUNX1), binding to the IL-17 promoter, and inducing FOXP3, in addition to inhibiting RAR-related orphan receptor γ2 (RORt), which is the transcription factor that regulates IL-17 [95]. The active form of vitamin D suppresses B cell growth, plasma cell differentiation, immunoglobulin production (IgG, IgM, and IgE), and memory B cell formation and causes B cell death [93].

### 4.14. Vitamin E

The daily dose of vitamin E recommended for adults is 15 mg. Many dietary fats and oils, particularly those high in polyunsaturated fatty acids (PUFAs), contain vitamin E; therefore, the dietary consumption of vitamin E is closely associated with the consumption of PUFAs [96]. Vitamin E is the most potent lipid-soluble chain-breaking antioxidant found in cell membranes and is thought to contribute to membrane integrity by reducing lipid peroxidation by ROS [97].

Several experimental studies performed in animals and humans have shown that vitamin E improves immunity by inhibiting COX2 activity, improving effective immune synapses in naive T cells, initiating T cell activation signals, and modulating the Th1/Th2 balance. These changes are primarily due to the reduced production of prostaglandin E2 (PGE2) because of inhibition of COX2 activity and decreased NO production [98]. In addition, vitamin E inhibits NF-κB activity, which is necessary for the maximum transcription of several proteins involved in inflammatory responses, including numerous cytokines, such as IL-1β, IL-2, and TNF-α that affect various inflammatory processes by decreasing vitamin E function [99]. The redistribution of key membrane-associated signaling molecules such as linker for activation of T cells family member 1 (LAT), the tyrosine-protein kinase ZAP70 (ZAP70), phospholipase C, and the Vav proteins, have also been proposed as a mechanism through which vitamin E enhances naive T cell function [100]. In addition to the suppression of PGE2, vitamin E supplementation among older adults has been found to decrease production of other pro-inflammatory markers such as TNF-α and IL-6, especially in response to infections [101,102]. Vitamin E supplementation decreases the inflammatory response in LPS-stimulated peripheral blood mononuclear cells (PBMCs) by decreasing pro-inflammatory cytokine production, such as TNF-α, IL-1, and IL-6, by monocytes [103].

### 4.15. Vitamin K

Vitamin K is a fat-soluble vitamin available in two natural forms, phylloquinone (K1) and menaquinone (K2), and a synthetic form, menadione (K3) [104]. An adult requires around 1 mg/kg body weight of vitamin K daily. Vitamin K derivatives (vitamin K_3_ and vitamin K_5_) decrease the proliferative response and cytokine release by activated T cells [105,106], and vitamin K derivatives suppress the production of IL-4, IL-6, IL-10, and TNF-α. Vitamin K supplementation also increases the numbers of CD4^+^, CD25^+^, and FOXP3^+^ Tregs [105].

According to the results of a mass spectrometric study, K3 may directly interact with the thiol antioxidant GSH and inhibit the activities of the extracellular signal-regulated kinase (ERK), JNK, and NF-κB in lymphocytes [106]. Due to the antioxidant effects of vitamin K3, NF-κB expression may be inhibited. NF-κB regulates the production of several cytokines and enzymes involved in immune responses [106]. In a meta-analysis, a synthetic analog of vitamin K_2_ was found to inhibit the development of secondary liver tumors, increasing the survival rate among patients with hepatocellular carcinoma; activation of apoptotic pathways and the suppression of NF-κB were proposed as possible mechanisms [107]. The key immunomodulatory roles played by vitamins are shown in Table 1.

### 4.16. Zinc

The recommended daily amount of zinc is 8 mg for women and 11 mg for adult men. Zinc plays a key role in the immunological response, and zinc-deficient individuals are more susceptible to a range of infections including malaria, tuberculosis, HIV, pneumonia, and measles. Zinc deficiency increases the vulnerability to infections via immunologic processes that have been studied for decades. Zinc appears to have a wide range of effects on the immune system, including effects on the physical skin barrier and gene regulation in lymphocytes. Zinc is also required for neutrophil and NK cell development and function, which are involved in the nonspecific innate immune system. In this regard, zinc deficiency impairs the development of adaptive immunity by inhibiting T lymphocyte expansion and the activation of Th1 cells, which produce cytokines and assist B lymphocytes [188,189]. Zinc deficiency also has an adverse effect on antibody production and B lymphocyte development, especially IgG synthesis. Macrophages, which play vital roles in many immunologic processes, are negatively influenced by zinc deficiency, resulting in the dysregulation of intracellular cell death, cytokine generation, and phagocytosis, among other functions. Zinc plays various roles in multiple fundamental cellular processes, including cell activation, cell division, DNA replication, and RNA transcription, and its effects on these important immunologic mediators are well understood [190].

**Figure 2:**
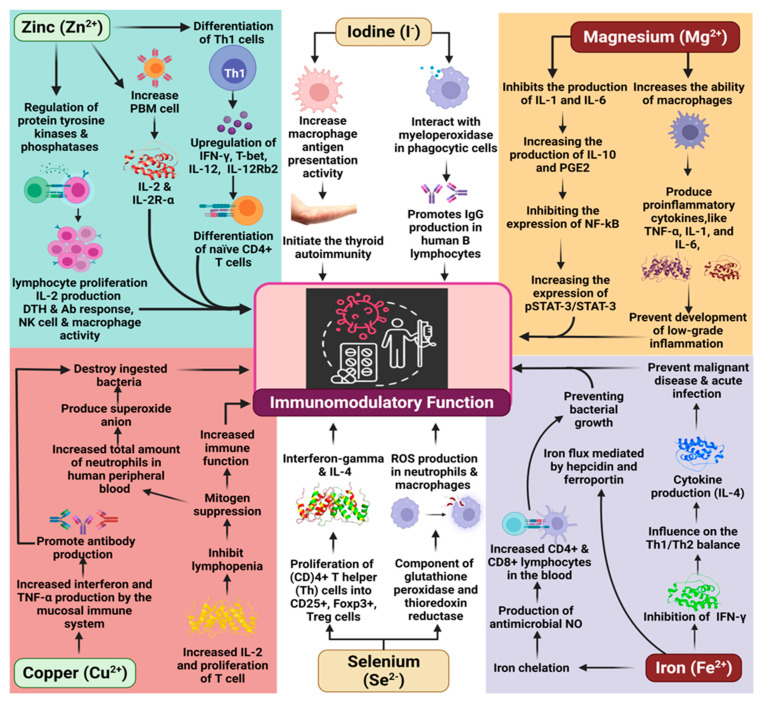
The key role of minerals as immunomodulators.

Research findings revealed that zinc promotes the development of tolerogenic phenotypes in bone-marrow-derived DCs, inhibiting MHC-II production, increasing the expression of the programmed death-ligand (PD-L) 1 and 2, and upregulating tryptophan degradation and kynurenine production, resulting in an imbalance between Th17 and T cells, which favors the proliferation of Tregs (Figure 2) [191]. Furthermore, zinc plays a pivotal role in the regulation of phosphatases (PTPs) and protein tyrosine kinases (PTKs). Several experimental studies have reported that zinc exerts an inhibitory effect on multiple PTPs, even at the very low amounts observed intracellularly [192]. Thymus involution, reduced Th1 cell numbers, and impaired immune functions, including lymphocyte proliferation, IL-2 production, delayed-type hypersensitivity responses, antibody responses, NK cell activity, macrophage phagocytosis, and specific neutrophil functions, are all impacted negatively by zinc deficiency [193].

### 4.17. Iron

The average daily allowance of iron is 13.7–15.1 mg for children, 16.3 mg for teenagers, 19.3–20.5 mg for men and 17.0–18.9 mg for women above 19. Iron is essentially required by all living organisms and is involved in a variety of biological processes. However, large amounts of free iron may be cytotoxic, when found in high amounts as it can stimulate the development of oxidative radicals that disrupt proteins, lipids, and nucleic acids. As a result, both iron deficiency and overabundance may confer detrimental impact on a wide range of cells, tissues, and organ functions; however, it has been challenging to relate such functional abnormalities to changes in particular iron-dependent biochemical pathways in general [194].

Heme is a vital component that can be found in abundance throughout our bodies’ tissues. The heme protein complex, which is made up of a variety of proteins, plays crucial roles in cellular physiology and metabolism [195]. Once free heme is liberated from cells and hemeproteins, it produces oxidative damage and inflammation, serving as a template for damage-associated molecular patterns in the body. This is because free heme is an important component of the pathogenic process of sterile and infectious hemolytic disorders such as malaria, hemolytic anemia, ischemia-reperfusion, and hemorrhage [196]. The mechanism through which heme produces ROS, activates innate immune cells, and triggers cell death is not well known [197]. In addition to increasing the formation of reactive oxygen species and inflammation-inducing mediators directly via its iron atom, heme may also activate certain signaling pathways and so indirectly enhance lipid peroxidation as well [197]. Heme stimulates innate immune cells such as macrophages and neutrophils by activating innate immune receptors on the surface of these cells [198].

Many of the proteins and genes involved in iron homeostasis also perform crucial roles in the regulation of iron flux, preventing bacteria from utilizing iron for growth. In addition, cells in the innate immune system, such as monocytes and macrophages, respond to bacterial infections by carefully regulating iron flux, mediated by hepcidin and ferroportin, which are expressed in microglia and lymphocytes. Several effector molecules, including TLRs, NF-κB, hypoxia-inducible factor-1 (HIF-1), and heme oxygenase (HO), regulate the inflammatory response by mobilizing various cytokines and neurotrophic factors [199].

The effects of iron on Tregs are much more nebulous and may be characterized as both direct and indirect effects. FOXP3, the master transcription factor in Tregs, appears to be inhibited by iron chelation, and iron chelation also reduces CD25 and signal transducer and activator of transcription 5 (STAT5) phosphorylation levels [200,201,202,203].

Iron also affects NF-κB activation. Normal to high iron levels induce the increased generation of ROS, which activate NF-κB and lead to the increased expression and release of cytokines. Under conditions of iron deficiency, ROS formation decreases, reducing the activation of NF-κB and cytokine release [204]. By contrast, iron chelation in macrophages increases the production of antimicrobial NO, which may help prevent infections. Total white blood cell counts, the proportion of CD4^+^ or CD8^+^ lymphocytes in the blood, the CD4^+^/CD8^+^ ratio, in vitro IL-2 production, CD8^+^ cytotoxicity, and delayed-type hypersensitivity responses are downregulated under conditions of iron overload (Figure 2) [204].

### 4.18. Selenium

The recommended dietary allowance (RDA) of selenium for adults aged 19 and above is 55 µg per day. Pregnant and breastfeeding women need between 60 and 70 micrograms per day, respectively. Selenium is an essential component of selenoproteins, which are necessary for NK cell, macrophage, neutrophil, and T lymphocyte functions [205]. Increased in vivo selenium levels significantly influence the proliferation and differentiation CD4^+^ Th cells into CD25^+^FOXP3^+^ Tregs [206,207]. Increased dietary selenium consumption results in the enhanced production of IFN-γ in response to T cell receptor (TCR) stimulation and the TCR-induced differentiation of CD4^+^ T cells, whereas reduced dietary selenium intake results in increased IL-4 production (Figure 2). Selenium levels influence B-cell-dependent antibody production in a pathogen-dependent manner, although these effects are less consistent than the effects observed on T cell immunity, according to the literature. B cell activation and differentiation are controlled by the oxidatively sensitive NF-κB and stimulate the production of leukotrienes, which may be influenced by selenium intake [207]. Inflammatory cytokine gene expression is regulated by NF-κB, and recent research suggests that decreased serum selenium levels contribute to NF-κB-mediated inflammation [208].

Selenium supplementation is commonly immunostimulatory, resulting in T cell proliferation, innate immune cell activation, NK cell activity, and a variety of other indicators [207]. Selenium supplementation also regulates the inflammatory response in patients with respiratory distress syndrome through the restoration of lung antioxidant activity, which lowers IL-1 and IL-6 levels, reducing the inflammatory response and considerably improving respiratory mechanics [209]. Selenium is an essential component of antioxidative enzymes, including thioredoxin reductase and glutathione peroxidase, and selenium deficiencies increase ROS generation in neutrophils and macrophages [210].

### 4.19. Iodine

The daily iodine allowance for males and non-pregnant women is 150 µg. For pregnant women, a daily dosage of 220 to 250 micrograms is suggested, whereas daily intake of 250 to 290 micrograms is recommended for breastfeeding mothers. Iodine contributes to the regulation of the immune system by fighting infection. It may improve immune function by assisting in the removal of both chemical and biological toxins. It also suppresses autoimmune responses, improving immunity control [211].

In vitro, iodine kills bacteria through interactions with myeloperoxidase in phagocytic cells and promotes IgG production by human B lymphocytes (Figure 2). Iodine deficiency is associated with an increased risk of immunodeficiency and cancer development due to disruptions in the antioxidant mechanism. Excessive iodine consumption is associated with both hypothyroidism and hyperthyroidism, which are characterized by decreased NK cell activity. In rats, increasing iodine concentrations increases macrophage antigen presentation activity, suggesting that iodine serves as an initiating factor in thyroid autoimmunity [212]. Thyroid autoimmunity is caused by excessive iodine intake, which causes an imbalance in cytokine production [213].

### 4.20. Magnesium

Magnesium is a trace element with a daily dietary requirement of 400–420 mg for men and 310–320 mg for women [214]. The protective effects of magnesium in inflammatory reactions are among the many functions associated with this vital mineral, which deserves special attention due to its importance. Magnesium deficiency increases the ability of macrophages to produce inflammatory cytokines, especially TNF-α, IL-1, and IL-6, resulting in the development of low-grade inflammation [215]. Pro-inflammatory cytokines are upregulated in HUVECs grown under low-magnesium conditions [216]. Magnesium also reduces the generation of TNF-α and IL-6 by LPS-stimulated cord blood mononuclear cells in vitro [217]. Mesenchymal stem cells (MSCs) have immunomodulatory properties that are modulated by magnesium, and magnesium inhibits the formation of IL-1 and IL-6 while increasing the production of IL-10 and PGE2 and inhibits the pNF-κB/NF-κB ratio while increasing the pSTAT-3/STAT-3 ratio [218]. Magnesium deficiency in rats causes cardiac dysfunction and inflammation, increasing the production of TNF-α, IL-1, and IL-6. When NF-κB is activated, magnesium regulates the production of cytokines, promoting disease development (Figure 2) [219].

### 4.21. Copper

Copper is an essential trace element with a daily dietary requirement of 900 µg for adults [220]. Copper has been well-established as involved in immune protective functionalities, supported by a large body of experimental evidence [3]. Copper plays a pivotal role as an important cofactor in redox reactions, regulating a wide range of physiological activities, including neurotransmission, energy production, connective tissue development, and iron metabolism [221]. Although a major portion of copper exists in the cupric (Cu^2+^) form, copper can readily donate and accept electrons, playing important roles in redox reactions and free radical scavenging [221]. The majority of copper in living organisms is closely connected to ceruloplasmin, rendering it inactive in Fenton-like processes. Free copper stimulates the production of free radicals when combined with biological reducing agents, according to some researchers. Inorganic copper in dietary supplements may raise free copper levels in the body, which may increase the risk of the production of reactive oxygen species, henceforth showing oxidative stress (referred to as prooxidant activity) [222]. Copper’s potential to act as a catalyst for the production of free radicals is well documented. Many in vitro studies have shown that copper (Cu^2+^) has been found to be a significantly more redox active metal than iron (Fe^3+^) in various applications [223]. Mild to severe copper deficiencies result in reduced IL-2 production and decreased T cell proliferation, resulting in lymphopenia, making copper an essential component for the maintenance of a healthy immune system [212]. Neutrophils in the peripheral blood decrease in cases of severe copper insufficiency, reducing the capacity to produce superoxide anions and destroy ingested bacteria [3].

In cattle, a copper deficit results in disrupted plasma cells, reducing antibody generation and decreasing the production of IFN-γ and TNF-α by the mucosal immune system. Increased copper administration is associated with a decreased proliferative response to concanavalin A in mice. Ex vivo studies indicated that increased copper levels in the blood are associated with lymphocyte inhibition and mitogen suppression, resulting in reduced immune function (Figure 2) [212]. Listed in Table 2 are key immunomodulatory roles played by minerals.

## 5. Conclusions and Future Directions

Boosting immunity represents the best approach to protecting hosts from various microbial attacks or other infections. Vitamins and minerals are important micronutrients involved in the interconnected and intricate immune system; however, only trace amounts are necessary to perform these functions. Our bodies cannot produce micronutrients, and many vitamins and minerals can only be obtained in varying concentrations through the ingestion of nutrient-rich foods. Various dietary sources can provide vitamins and minerals, which support and strengthen our immune systems. Vitamins and minerals often have synergistic effects throughout the various immune system stages and compartments. Furthermore, vitamins and minerals can both stimulate and suppress immunological responses, and enhance the proper operation of the immune system by providing antioxidants that reduce cell damage caused by free radicals and assist in T cell synthesis. Vitamin and mineral deficiencies can lead to inadequate generation of important immune components including both cellular and molecular components, resulting in ineffective immune responses to pathogenic factors.

Numerous studies report that the right balance of vitamins and minerals is vital for developing an efficient immune response that can minimize the risks associated with viral and bacterial infections. However, in both animal and human models, dietary components may enhance or diminish the response of the immune response. Moreover, immunomodulation can vary from person to person and animal to animal. The underlying mechanisms of action remain uncertain, particularly under diseased conditions, and clinical investigations regarding the immunomodulatory influence of vitamins and minerals remain necessary. Further studies are necessary to unravel the immunomodulatory influences of food components, including the establishment of optimal vitamin and mineral dosages to promote immune health. Sufficient evidence regarding the immunomodulatory effects of vitamins and minerals can support the development of preventative and curative treatments for a variety of autoimmune disorders (lupus, rheumatoid arthritis, and multiple sclerosis), infectious diseases, and other allergenic conditions.

## Figures and Tables

**Table 1:** The immunomodulatory roles of vitamins, reported by experimental studies.

Vitamins	Dose/Concentration	Study Model	Findings	References
Vitamin A	500 nM	In vitro (peripheral blood mononuclear cells)	RA inhibits IFN-γ, IL-17, and IL-9 production in CD4+ T cells, upregulates CD103 expression and gut-homing receptors	[44]
	0.1 μM	In vitro (peripheral blood mononuclear cells)	RA prevents human nTregs from converting to Th1 and/or Th17 cells and sustains their Foxp3 expression and suppressive function, suppresses IL-1 receptor (IL-1R) upregulation, accelerates IL-6R downregulation	[45]
	0.01 M	In vitro (myelomonocytic cells)	Retinoids inhibit mumps virus in vitro due to upregulation of type I interferon (IFN) and IFN stimulated genes	[108]
	Concentration in olive oil suspension 25 mg/mL and inject intraperitoneally 125 μg/gm weight	In vivo (Mice)	Retinoic acid acts intrinsically in developing gut- tropic premucosal dendritic cell, directs generation of intestine-like cDC1 and cDC2 subsets	[109]
	retinyl acetate (25 µg/d)	In vivo (Mice)	Effective for clearance of *Citrobacter rodentium*, fewer CD8αβ+ cells and more CD8αα+ and T cell receptor (TCR)γδ+ T cells, enhanced IL17	[110]
	5 or 25 nM	In vitro (Human tonsillar B cells)	Increases IgA production, the expression of germ-line IgA1, IgA2 transcripts (GLTa1 and GLTa2), and the frequency of IgA1-secreting B cell clones, and induces IgA isotype switching	[111]
	1 µM retinol	In vitro (Murine respiratory tract lung epithelial cell)	Increases IgA production by lipopolysaccharide (LPS)-stimulated splenocytes cultures, and increases the expression of MCP-1, IL-6, and GM-CSF	[112]
	Control diet of retinol palmitate 15 IU/g	In vivo (Mice)	Causes reduction of immunodominant CD8+ T cell frequencies in the lower respiratory tract (LRT) airways of VAD animals, T cells, and shows unusually high CD10	[113]
	1 μM	In vivo (mouse splenocytes)	Lowers Th17 T-cell activity, downregulating IL-6, promoting B cell production of IgA, upregulating IL-6, and increasing transcript levels of MCP-1, GMCSF, and IL-10 in MACs	[114]
	0 or 15 IU g^−1^ vitamin A	In vivo (Mice)	Increases cytokine/chemokine gene expression and cytokine protein production in VAD animals. Viral infection persists longer in the upper and lower respiratory tract of VAD mice	[115]
	250 µg per day	In vivo (transgenic mice)	Regulates the homeostasis of pre-Dendritic cell (DC)–derived DC subsets and have implications for the management of immune deficiencies	[116]
	0 or 15 IU/g vitamin A palmitate	In vivo (Mice)	VAS decreases death and diarrhea-related mortalities in children, and increases immune responses toward pediatric vaccines	[117]
	0 to 25,000 IU kg^−1^	In vivo (Mice)	Controls local lymphoid tissue inducer cell differentiation and maturation upstream of the transcription factor RORct	[118]
	10 nM RA	In vivo (Mice)	Abrogates RA signaling in B cells; these cells were not able to induce a4b7 expression. RA signaling in B cells is important for the induction of IgA+ GC, effective gut humoral response, and to maintain a normal microbiota composition. It has a direct effect on IgA plasma cell differentiation	[119]
	60 mg/kg	In vivo (Mice)	Lowers interferon (IFN)-g production, activates NKT cells, decreases extracellular signal-regulated kinase (ERK) phosphorylation, and enhances phosphatase 2A (PP2A) activity	[120]
Vitamin C	2 mg/kg, 150 mg/kg	In vivo (piglets)	Decreases vulva length, width, height, and area,. Decreses the concentrations of BUN, CRE, AST and TBIL in serum, and reduces IgA, IgG and IgM levels. Restoring serum estradiol (E2), progesterone (PROG), luteinizing hormone (LH) and follicle stimulating hormone (FSH) levels in weaning piglets	[121]
	50 to 200 mg/kg/24 h.	In vitro (venous blood sample)	Increases the expression of the antimicrobial proteins, ↓MtDNA levels	[122]
	200 mg/kg	In vivo (Mice)	Decreases lung neutrophil extracellular traps, decreases circulating free-DNA following peritonitis-induced sepsis. In vitamin C deficient neutrophils, Upregulates endoplasmic reticulum stress associated gene expression, Induces autophagy signaling and increases PAD4 mRNA	[123]
	162 ± 8 mg vitamin C per 100 g fruit	In vitro (venous blood)	No effect on control (non-stimulated) neutrophil migration, increases neutrophil oxidative burst, improvement of important neutrophil functions	[124]
	57 mg/kg/day	In vivo (Mice IRI)	Lowers the severity of tubular injury and renal arterial resistance	[125]
	100 nM	In vitro (peripheral blood mononuclear cells)	Increases oxidative stress, lowers proinflammatory mediator, ROS, TNF-α,and IL-6, LPS-Induced Autophagy in MH-S Cell Lines	[126]
	200 mg/kg	In vivo (Mice)	Prevents sepsis-mediated disassembly of the Na+-K+-ATPase pump, causes cytoskeletal rearrangements, changes in viscoelastic properties, increases epithelial ion channel and transporter expression, and increases alveolar fluid clearance	[127]
	3.3 g/L	In vivo (Mice)	Increases type I interferons (IFNs), increases IFN-α and -β, increases infiltration of inflammatory cells (IL-1α/β and TNF-α) into the lung	[128]
Vitamin D	1 to 100 nM	In vitro (peripheral B cell line)	Increases IgE production by 63.9 ± 5.9%., decreases CD19+ CD27high CD38+ B cell population, and decreases AID expression	[129]
	0.1 μM, 1.0 μM, 10 μM	In vitro (human PBMCs)	Lowers T helper (Th) cell population-specific cytokine expression of interferon γ (Th1), interleukins IL-17A (Th17) and IL-22 (Th17/Th22), ↑IL-4 (Th2) levels	[130]
	(6 µg/kg/day in PBS/0.03% ethanol)	In vivo (Mice)	Lowers the IgE response in a type I allergy mouse model	[129]
	n.m	In vitro (PBMCs)	Causes inhibition of IgE production by calcitriol is mediated by its transrepressive activity through the VDR-corepressor complex	[131]
	0.05–50 mg/kg 25(OH)D	In vivo (mice)	Decreases the allergic airway inflammation, Th2 cytokine expression in the lungs. And the humoral immune reaction	[132]
	0–50 nM	In vivo (Mice) and human PBMCs	Decreases CD8 and CD4 T cells proliferation, and IL-2, ↓IFN-γ and ↓IL-17 production, increases IL-4 and IL-10 production and human Treg development	[133]
	25(OH)cholecalciferol (40 ng/mL) or 1,25(OH)2cho- lecalciferol (20 ng/mL)	In vitro (peripheral blood mononuclear cells)	Lowers interleukin (IL)-9, IL-5, and IL-8. Increases IL-13þ cells, downregulates transcription factors PU.1 and interferon regulatory factor 4	[134]
	10^−9^ M, 10^−8^ M or 10^−7^ M	In vitro (Primary bronchial epithelial cells)	Causes suppression of viral replication, and increases LL-37 expression	[135]
	5(OH)D(<50 nmol/L, 50–75 nmol/L ≥75 nmol/L)	In vitro (peripheral blood mononuclear cells)	Reduces systemic inflammation	[136]
	100, 10 or 1 ng/mL of vitamin D3	In vitro (human monocytes)	Upregulates the expression of NK cytotoxicity receptors NKp30 and NKp44, as well as NKG2D, downregulates the expression of the killer inhibitory receptor CD158, and downregulates the expression of CCR6 on the surface of iDCs	[137]
	10 nM 1,25D3	In vitro (peripheral blood mononuclear cells)	inhibits the cytokine response of CD40L-stimulated macrophages, decreases CD40L-induced expression, decreases tumor necrosis factor (TNF)-α production, decreases interleukin (IL)-1β production, and (IFN)- γ proliferation, and increases IL-10 production	[138]
	Vitamin D3 (cholecalciferol) at >2000 IU/kg	In vivo (Mice)	1,25(OH)2D3/VDR-dependent induction of IL-10 production can contribute to the mast cell’s ability to suppress inflammation and skin pathology at sites of chronic UVB irradiation	[139]
	four fortnightly doses of 2.5 mg vitamin D3	In vitro	Increases chemokines (AMP MMP-9) and antigen-stimulated Th1 cytokine suppression, decreases IL-4, CCL5, and IFN-α secretion, accelerates sputum smear conversion, and enhances treatment-induced resolution of lymphopenia, monocytosis, hypercytokinemia, and hyperchemokinemia	[140]
	10^−8^ M	In vivo (Mice)	Restrains the inflammatory response of NOD and C57BL/6 BM-derived DCs, decreases secretion of CCL3, CCL4, CCL5, and CXCL10, increases secretion of CCL2 and CCL7, decreases T cell stimulatory capacity, and increases migration-competent tolerogenic DCs	[141]
	1 μg/kg VD	In vivo (Mice)	Upregulates p53 acetylation-mediated apoptosis in MH7A cells, promotes Sirt1 translocation and apoptosis of FLSs	[142]
	100/30 nM	In vitro (Human alveolar epithelial cell line)	Decreases autophagy, enhances apoptosis, decreases H1N1-induced TNF-α level, IFN-b (interferon-beta), and IFN-stimulated gene-15. Downregulates IL-8 as well as IL-6 RNA levels and suppresses the H1N1- induced transcription	[143]
	10 or 100 nM	In vitro (peripheral blood mononuclear cells)	Causes suppression of NK cytotoxicity and downregulation of CD107a expression in NK cells, increases CD158a and CD158b expression, decreases the expression of NKp30 and NKp44 on CD56+CD3− NK cells, and lowers CD56+/IFN- γ+ and CD56+/TNF-α+	[144]
	100 nmol	In vitro (bronchial epithelial cell line)	Decreases rhinovirus replication and release, and increases rhinovirus-induced interferon stimulated genes and cathelicidins	[145]
	100,000 IU of cholecalciferol per week for 4 weeks, followed by 100,000 IU of cholecalciferol per month for 6 months	In vitro (SLE patients)	Increases naïve CD4+ T cells and regulatory T cells, decreases effector Th1 and Th17 cells, and decreases memory B cells and anti-DNA antibodies	[146]
	5 ng/mL	In vitro (peripheral blood mononuclear cells)	Increases Foxp3+ and IL-10+ CD4+T cells	[147]
Vitamin E	TRF supplements (200 mg each capsule) 400 mg per day	In vitro (human blood leukocytes)	Enhances production of IFN-y, f IL-4, IL-6, and TNF-α	[148]
	50 mg/d	In vivo (Human)	Decreases incidence of pneumonia by 69%	[149]
	1 kg of T3 supplemented diet (1 g Tocomin 50% +39 g vitamin E- stripped soybean oil + 0.96 kg basal die)	In vivo (Mice)	Increases splenocyte IL-1b production, Lymphocyte proliferation, tumor necrosis factor-a, and interferon-ꭚ	[150]
	50–200 μg /mL	In vitro (human PBMCs)	Increases cAMP production, IL-2 production, IL-17, IL-8, and RANTES	[151]
	30 or 500 ppm of vitamin E (RRR-a-Tocopheryl acetate)	In vivo (Mice)	Gamma-T was more effective but less specific than alpha-T in the presence of vitamin E; CD40L is strongly upregulated by alpha-T, but down-regulated by gamma-T, gamma-T appears to better prevent e induction of gene expression upon T cell stimulation, e.g. of some cytokines (interleukin 3 and 10) and chemokines (chemokine ligand 9, 10, and 11)	[152]
	250 to 500 mg D-α-tocopherol/kg diet	In vivo (Mice)	Decreases lung CD11b+ dendritic cell subsets, lung mRNA expression of IL-4, IL-33, TSLP, CCL11, and CCL24	[153]
	30 (control) or 500 (supplemented) ppm of vitamin E	In vivo (Mice)	Increases the expression of genes (Ccnb2, Cdc2, Cdc6) in old T cells. Increases upregulation of IL-2 expression, decreases upregulation of IL-4, has impact on signal transduction, transcriptional regulation, and apoptosis pathways in T cells	[154]
	46 mmol/L of vitamin E	In vitro (Mice spleen cells)	Vitamin E eliminates the age-related differences in LAT phosphorylation in both T cell subsets, and difference in the tyrosine phosphorylation of LAT	[155]
Vitamin K	vitamin K (1–5 μM)	In vitro (Bone marrow-derived macrophages)	Causes inhibitors of the NLRP3 inflammasome to block the interaction between NLRP3 and ASC, which attenuates the severity of inflammation	[156]
	MK-4 (0.5–20 μmol/L)	In vitro (microglial cell line (BV2))	Suppresses the upregulation in the expression of iNOS and COX-2 in the cells and the production of TNF-α and IL-1β. Inhibits ROS production, p38 activation, and rotenone-induced nuclear translocation of NF-κB in BV2 cells	[157]
	20 mM menadione	In vitro (Mice Splenocytes)	Increases ROS levels, thus suppressing production in lymphocytes and CD4 + T cells, and activation of ERK, JNK and NF- κB	[158]
	Vitamin K2	In vitro (human bladder cancer cell lines)	Induces apoptosis in bladder cancer cells, generates reactive oxy- gen species (ROS), phosphorylating of c-Jun N-terminal kinase (JNK) and p38 MAPK	[159]
	0.1–100 μM	In vitro (PBMCs)	Suppressing the mitogen-activated proliferation, inhibiting the production of tumor necrosis factor (TNF) α, interleukin (IL)-4, -6, and -10, and increases Treg cells	[160]
Vitamin B1	(0 µg/mL, 0.125 µg/mL, 0.25 µg/mL, 0.5 µg/mL, 1 µg/mL, and 2 µg/mL)	In vitro (breast epithelial cells from non-cancer origin and metastatic site)	Reduces extracellular lactate levels, increases cellular pyruvate dehydrogenase (PDH) activities, decreases non-glycolytic acidification, glycolysis, and glycolytic capacity, and reduces cell proliferation in MCF7	[161]
	Not mentioned	In vitro (Mice encephalitogenic cells)	TD aggravated the development of EAE, causing microglial activation, increases leukocyte infiltration in the spinal cord, Th1, and Th17 cells, and upregulates expression of CCL2	[162]
	complete chow (303.3 ± 42.6 nmol/L) thiamine deficiency	In vivo (Mice)	Thiamine deficiency increases TNF-α and MCP-1 concentrations, decreases blood IL-1β level, and increases KC, IL-1 β, and IL-6	[163]
Vitamin B2	low (3·1 nM), physiological (10·4 nM) or high (300 and 531 nM)	In vivo (mouse monocyte/macrophage cell line)	Low riboflavin content decreases the proliferation rate and increases apoptotic cell death, completely inhibits the respiratory burst and slightly impairs phagocytosis, and impairs cell adhesion	[164]
	3.1 nM to 10.4 nM	In vivo (Mycoplasma-free mouse preadipocytes)	Riboflavin deprivation Induces adipocyte death, increases lipolysis and free fatty acid release, ROS Production, NF-κB Phosphorylation, and pro-inflammatory TNFα and IL-6	[165]
	1 µg/mL, 0.5 µg/mL, 0.25 µg/mL, 0.125 µg/mL, 0.62 µg/mL, 0.31 µg/mL, 0.15 µg/mL and untreated control 0 µg/mL for vitamin B2	In vitro (U937 cell line)	Inhibits cell migration of pro-Monocytic cells, decreases the expression of PD-L1, increases secretion of IL-8 and IL-10, and increases GM-CSF	[166]
Vitamin B3	100 mg/kg/d niacin	In vivo (guinea pigs)	Downregulates inflammatory factors (IL-6 and TNF-α), suppresses protein expression of CD68 and NF-κB p65, attenuates oxidative stress	[167]
	10^−3^–10^−6^ M Nicotinic acid	In vitro (preadipocytes cells)	attenuating expression of fractalkine, MCP-1, RANTES, iNOS, and macrophage chemotaxis	[168]
	Niacin (0–1 mM)	In vitro (human aortic endothelial cells)	Inhibits production of ROS, LDL oxidation, TNF-α, NF-κB activation, and vascular cell adhesion molecule-1 (VCAM-1)	[169]
	Nicotinic acid (0.1–3 mM)	In vitro (human monoblastic leukemia cell line)	Induces macrophage PGD2 secretion	[170]
	80 and 320 mg/kg	In vivo (rats)	Increases colonic MPO activity and TNF-α level, and decreases cytokine IL-10	[171]
	100 mg and 250 mg niacin	In vivo	Causes macrophage polarization from M1 (pro-inflammatory) to M2 (counter-inflammatory), boosting anti-inflammatory processes, thus suppressing inflammation	[172]
	niacin (1, 10, 100 μmol/l)	In vitro (Murine alveolar macrophages)	reduces the levels of TNF-α, IL-6 and IL-1β in LPS-challenged alveolar macrophages, inhibits NF-κB activation, attenuates the LPS-induced pro-inflammatory cytokines	[173]
	niacin (0.25–1 mM)	In vitro (umbilical vein endothelial cells)	Decreases IL-6 and TNF-α secretion and inhibits NF-κB p65 and notch1 protein expression	[167]
Vitamin B5	10 µM	In vivo (C57BL/6J mice)	significantly inhibits the growth of *Mycobacterium tuberculosis* by regulating innate immunity and adaptive immunity	[62]
Vitamin B6	(1000 µg/mL, 500 µg/mL, 250 µg/mL, 125 µg/mL, 62.5 µg/mL, 31.25 µg/mL, 15.6 µg/mL	In vitro (U937 cell line)	Inhibit cell migration and proliferation of pro-monocytic cells, decreases the expression of PD-L1 and IL-1β, and increases secretion of IL-8 and IL-10	[166]
	0, 12 mg, and 120 mg per kg diets	In vivo (male BALB/c mice)	Vitamin B6 deficiency decreases growth rate, lymphocyte proliferation, and CD4 T Lymphocyte Differentiation	[66]
	5, 10, 20, and 40 mg/kg	In vivo (Rex rabbits)	Increases IL-2, IFN-γ, M cell number, weight of thymus and spleen, cell division, prolifferation, and maturation	[68]
	100 nM PMA, B6 vitamer (500 µM)	In vivo (Mice)	Vitamin B6 inhibits LPS-induced NF-κB, JNK activation, and gene expression, suppresses NLRP3 inflammasome activation, noncanonical IL-1β secretion and pyroptosis, and inhibits signal 1 and signal 2 for the IL-1β production	[69]
Vitamin B7	0.8 mg d-biotin per kg	In vivo (mice)	Biotin deficiency upregulates TNF-α production; however, no differences were detected in NF-κB activity	[174]
	basal diet (0.8 mg/kg of d-biotin) biotin deficient (biotin free)	In vivo (Mice)	Improves Ni allergy, increases PUFA in rat liver, decreases IL-1b production and proliferation, and decreases TNF-α production	[175]
	Control (d-biotin 0.2 mg/L) BD (biotin free)	In vitro (murine macrophage cell line)	Inhibits augmentation of IL-1b production	[175]
	0, 10 and 100 µM biotin	In vitro (Human dendritic cells)	BD enhancing TNF-α, IL-12p40, IL-23, and IL-1β secretion, and IFN-γ and IL-17 induction	[176]
Vitamin B9	(2 mg/kg diet)	In vivo (Mice)	Decreases total T cells and NK cell, increases TNFα immunoreactive protein, and increases liver Abca1 mRNA	[177]
	0.2 to 25 ppb	In vivo (Mice)	In vitro, vit-B9 reduces condition differentiated Treg cells from naïve cells but fails to survive. In vivo, depletion of vit B9 results in reduction of Treg cells in small intestine	[178]
	(1000 µg/mL, 500 µg/mL, 250 µg/mL, 125 µg/mL, 62.5 µg/mL, 31.25 µg/mL, 15.6 µg/mL and	In vitro (U937 cell line)	Exerts an anti-tumorigenic effect, inhibits cell migration, proliferation of Pro-Monocytic Cells, lowers the expression of PD-L1, and increases secretion of IL-8 and IL-10	[166]
	5 mg Folic Acid	In vivo	Increases UMFA concentrations and decreases the number and cytotoxicity of NK cells	[179]
	control medium (100 ng/mL) low folate medium (10 ng/mL) and no folate medium	In vitro (mouse monocyte cell line)	Folate deficiency enhances the pro-inflammatory cytokine increase in pro-inflammatory marker expression, blunts the generation of nitric oxide in response to an LPS challenge	[180]
Vitamin B12	0.3 μM	In vivo (Mice)	B12 lessens kidney IRI, decreases kidney inflammation and fibrosis, DNA damage response (DDR) and apoptosis, and hypoxia/reperfusion (H/R) injury, and modulates H/R induced chemokine gene expression	[181]
	2–64 μM	In vitro	Exerts robust protection against 30 μM concentrations of the pro-oxidants homocysteine and hydrogen peroxide, inhibits intracellular peroxide production, and prevents apoptotic and necrotic cell death	[182]
	low (<250 pg/mL) and normal (>250 pg/mL) vitamin B12	In vitro (PBMCs)	Low vitamin-12 level increases production of IL-6, IFN-γ	[183]
	10–50 nM	In vitro (Primary HAEC)	Prevents homocysteine-induced increases in O_2_•− and cell death, homocysteine and rotenone-induced mitochondrial O_2_•− production	[184]
	Not mention	In vivo (Mice)	Increases CD8+ T cells, decreases CD4/CD8 ratio, and decreases T reg in control groups than pre and post treatment groups.	[185]
	Not mention	Cross- sectional study	Negatively associated with TNF-α, HOMA-IR, serum resistin (children) and positively associated with pro-inflammatory cytokines and biochemical markers	[186]
	300% of the daily intake	In vivo (rats)	Decreased negative impact of protein malnutrition facilitates the production of T lymphocytes	[187]
	1000 µg/day intramuscularly	In vivo	Restoring increased CD4/CD8 ratio and depressed NK cell activity, increases C3, C4, and immunoglobulins	[75]

**Table 2:** The immunomodulatory roles of minerals reported by experimental studies.

Minerals	Dose/Concentration	Study Model	Findings	References
Zinc	91 mg/kg	In vivo (mice)	Increases Treg cells, decreases severity of EAE, and decreases Th17 RORγT+ cells	[224]
	6.77 μM	Pro-inflammatory TNF-α and IL-6	Causes zinc depletion, decreases production of TNF-α and IL-6, and increases phagocytosis and oxidative burst	[225]
	10 mg/day for 10 days	In vitro (PBMCs)	Decreases Th1-cytokine production and proliferation in MLC, prolongs Foxp3 expression, causes Sirt-1 inhibition and induces regulatory T cells in MLC	[226]
	10 μM, 20 μM, or 45 μM zinc sulfate/pyrithione (50 μM)	In vitro (PBMCs)	Suppresses activation of IκB kinase β (IKKβ) and NF-κB TNF-α release and subsequent TNF-α production. Contributes to anti-inflammatory action of PDE inhibitors	[227]
	2 mM Zn^2+^ and 2 mM PT	In vitro (Vero-E6 cells)	Inhibits the RNA-synthesizing activity of the RTCs and blocks the initiation step of EAV RNA synthesis	[228]
	1 μM + 10 μM pyrithione	In vitro (PBMCs)	Reduces mRNA expression of proinflammatory cytokines, and inhibits LPS-mediated toxicity	[229]
	zinc-deficient diet (0.5–1.5 ppm zinc) or a matched control diet (50.5–51.5 ppm zinc)	In vivo (mice)	Zinc-deficient dietary intake causes excessive inflammation to polymicrobial sepsis in conjunction, ZIP8 is a potent negative regulator of NF-kB activation	[230]
Iron	10, 50 or 100 μM	In vivo (mice)	Dietary iron loading lowers inflammatory responses such as Il-1β expression, decreases M1 marker, CD86, and I-A/I-E expression, increases IL-4, ↓NF- κB p65 nuclear translocation, and decreases iNos and pro-inflammatory cytokines expression	[231]
	250 μM	In vivo (diabetic mice)	Dietary iron overload causes hepatic oxidative stress and NLRP3 inflammasome activation, increase in hepatic inflammatory mediators and immune cell activation, upregulation of chemokine, cytokine, and antioxidant mediators such as iNos, TNF-α, Mcp1, hepcidin (gene name Hamp), Hmox1, and Tlr4	[232]
	(0.2–10 mg iron/kg; 0.2 mL/mouse)	In vivo (mice)	Decreases CD3+ and F4/80+ cells, decreases DTH reactions, and IFN-γ production, increases IL-4 production, and decreases splenic CD11b+ cells	[233]
	ferumoxytol (2.73 mg Fe mL^−1^, 8.37 mg Fe mL^−1^	In vivo (mice model)	Inhibits growth of subcutaneous adenocarcinomas Increases the presence of pro-inflammatory M1 macrophages in the tumor tissues	[234]
	ferumoxytol (0–30 mg mL^−1^)	In vitro (mammary tumor cells)	Iincreases caspase-3 activity, increases mRNA associated with pro-inflammatory Th1-type responses, Increases production of tumor-necrosis factor-α (TNF-α)	[234]
	FAC (0∼400 μg/mL)	In vitro (RAW264.7 macro-phage cell line)	Decreases mRNA levels of IL-6, IL-1, TNF-α, and decreases iNOS production	[235]
Selenium	0.08 to 1.00 mg/kg	In vivo (mice)	Increases CD4+ T cell responses and differentiation, increases Ca2+ mobilization, oxidative burst, and NFAT translocation, increases IL-2 transcription, IL-2 receptor expression, and proliferation	[236]
	with or without 0.2 ppm Se	In vivo (mice)	Mice fed a Se-deficient diet have more adult worms than Se-sufficient diet. Se-sufficient diet increased Il4, Il13, and Il13ra2 expression and decreased Il4 and Il13 expression. Restores anti-fecundity response	[237]
	100 nmol/L Se	In vitro (BMDM culture)	Increases Arg-I, Fizz1, and Mrc-1 expression, decreases TNF-α and IL-1β expression, produces endogenous activators to mediate the PPARg-dependent switch from M1 to M2 phenotype and participate in wound healing and inflammation	[238]
	MSA (2.41 μg/mL, 1.5 μg/mL Se) and MSC (4.15 μg/mL, 1.5 μg/mL Se)	In vitro (S. aureus Culture)	Displays a greater defense against uterine inflammatory damage, decreases necrosis factor alpha (TNF-α) and increases interleukin-6 (IL-6), ↓phosphorylation of IκBα and ↓NF-κB p65	[239]
	MSA (0 to 30 μM)	In vitro (DLBCL cell lines)	Inhibits HDAC activity, acetylation of histone H3	[240]
	0.03 to 1.5 mg/kg	In vivo (mice)	Se-deficient mice increase TNF-α, IL-1β, and IL-6 production, increase mRNA and protein expressions of toll-like receptor 2 (TLR2)	[241]
	100 μg/day	In vivo (mice)	Increases IFN-γ and IL-12 production, higher survival rate, and DTH response	[242]
	0.1 ppm	In vivo (mice)	↑interferon-γ, ↑interleukin IL-6	[243]
	100 μg/kg	In vivo (mice)	↑CD4+ CD25+ Foxp3+ T cells, ↑Foxp3 mRNA expression	[244]
	200 μg of Se	In vitro (PBMCs)	↑Interleukin (IL-2, IL-4, IL-5, IL-13, and IL-22)	[245]
Magnesium	60 mg/Lor 2.5 mM	In vitro (PBMCs)	↓production of TNF-α and IL-6 in maternal and neonatal, ↓cytokine production, ↓NF-kB activation, ↑constitutive IkBa level	[219]
	5 nM to 20 nM	In vivo (murine)	Inhibits activation of macrophage, ↓percentage of CCR7-positive cells, ↓cytokines (IL-1β, IL-6 and IL-10), ↓nuclear translocation and phosphorylation of nuclear factor-κB (NF-κB), ↑chondrogenic differentiation of hBMSCs	[246]
	Mg^2+^ (0, 1, 3 and 5 mM)	In vitro (murine MSCs cells)	↑proliferation rates of MSCs, ↓IL-1β and IL-6, ↑IL-10 and PGE2, ↓pNF-κB/NF-κB, ↑pSTAT-3/STAT-3, modulating the production of IL-1β and IL-6	[218]
	concentrations of Mg- supplemented (0.8, 5, 10, 15, and 20 mmol/L)	In vitro (asthmatic CD4+T cells)	↓IL-5 and IL-13 secretion, ↑IFN-y secretion, modulating the immune responses of acute asthmatic CD4* T cells	[247]
	499 mg/kg and 44 mg/kg in the control diet and the low-Mg diet, respectively	In vivo (rat)	Mg deficiency increased the levels of mRNA known to be expressed by mast cells in the liver; mast cells were locally distributed around portal triads	[248]
	32 and 950 mg/kg respectively for deficient and control diets	In vivo (rats)	In Mg deficient diet ↑interleukin-6 (IL-6) level, higher u2-macroglobulin and u1-acid glycoprotein, ↑plasma fibrinogen and ↓ albumin concentration	[249]
Copper	1 µm Cu group, 200 mg/kg CuCl_2_ group, 200 mg/kg nano-Cu low group, 50 mg/kg nano-Cu medium group, 100 mg/kg nano-Cu nano-Cu high group, 200 mg/kg	In vivo (rat)	Decreased antibody production (IgA, IgG, IgM) altered lymphocyte subpopulation in the spleen, altered the number of blood cells, induces oxidative stress	[250]
	20–1000 mg/kg DW nCu- treated seeds (nCu-1000); 2–1000 mg nCu/l-treated mice	In vivo (mice)	Administration of mice with 1000 mg/l nCu leading to inflammatory responses, upregulated expression of serum biochemical indicators of liver and kidney, increased infiltration and activation of splenic immune cells	[251]
	163 mM/L copper sulfate	In vitro (stromal cell)	Copper disrupted the endometrial receptivity signature of dHESCs, decreases IGFBP1 levels, does not increase the apoptosis level	[252]
	163 mM/L copper sulfate	In vitro (endometrial cells)	Changes in the gene expression (42 up- and 9 downregulated), does not increase the apoptosis level induced by the decidualization treatment	[252]

## Data Availability

Available data are presented in the manuscript.
